# From touch to triage: translating the NAME model into clinical practice for enhanced neonatal assessment

**DOI:** 10.3389/fped.2026.1734450

**Published:** 2026-03-04

**Authors:** Francesco Cerritelli, Caterina Accardi, Alessia Alati, Adele Alberti, Marco Chiera, Matteo Galli, Chiara Leva, Erica Lombardi, Micol Pivotto, Sonia Travaglini, Sonia Zanini, Jordan Keys, Kimberly Wolf, Andrea Manzotti

**Affiliations:** 1 NYIT College of Osteopathic Medicine, Old Westbury, NY, United States; 2 RAISE Lab, Foundation COME Collaboration, Milan, Italy; 3 Research Department, SOMA, Milan, Italy; 4 Touro University California College of Osteopathic Medicine, Vallejo, CA, United States; 5 Osteopathy’s Promise to Children, San Diego, CA, United States; 6 Division of Neonatology and Neonatal Intensive Care Unit, Buzzi Children’s Hospital, Milan, Italy; 7 Vita-Salute San Raffaele University, Milan, Italy

**Keywords:** autonomic nervous system, neonatal assessment, NICU clinical tools, prematurity and health outcomes, touch-based evaluation

## Abstract

**Background/objectives:**

The Neonatal Assessment Manual scorE (NAME) model has emerged as a novel, structured, touch-based approach to evaluating neonates’ general conditions, with growing evidence supporting its validity and reliability in NICU settings. However, there is a critical need to integrate this method into clinical workflows and explore its translational potential in improving neonatal care. This paper aims to consolidate the body of evidence surrounding the NAME model and propose a clinically implementable strategy to enhance neonatal assessment, early detection of complications, and overall health outcomes in NICU settings.

**Methods:**

We critically appraised key NAME studies encompassing theoretical rationale, construct and content validity, inter-rater reliability, and clinical correlations in NICU populations. Drawing from these findings, we developed a stepwise clinical framework for NAME integration, aligning it with existing neonatal care protocols.

**Results:**

Evidence demonstrates that NAME scores correlate significantly with infants’ gestational age, birth weight, and complexity indices (*p* < 0.001), providing a rapid and non-invasive method to stratify newborns’ health conditions. Inter-rater reliability is moderate-to-good, particularly for “Marginal” classifications, and professionals across NICU disciplines found the method to have high content validity (CVI ≥ 0.9). A structured roadmap for clinical integration is proposed, including operator training guidelines, NAME score interpretation algorithms, and embedding NAME within multidisciplinary rounds.

**Conclusions:**

The NAME model, grounded in physiological and clinical evidence, represents a promising paradigm shift in neonatal assessment. Its systematic adoption may facilitate early risk detection, personalized care planning, and improved outcomes in NICU populations. Future implementation studies are needed to validate its operational impact across diverse care settings and age groups.

## 1 Introduction

Preterm birth remains a significant global health concern, with over 15 million babies born preterm (<37 weeks gestational age) annually, accounting for nearly 11% of all live births worldwide (1). It is the leading cause of neonatal mortality and a major contributor to long-term neurodevelopmental impairment, including cognitive, sensory, and motor deficits (2). Even late-preterm and full-term infants admitted to neonatal intensive care units (NICUs) often experience complications related to perinatal stress, maternal health, or birth trauma, necessitating continuous and nuanced clinical evaluation (1, 2).

Despite substantial technological advancements in neonatal monitoring—such as cardiorespiratory telemetry, cerebral oximetry, and point-of-care ultrasonography—the clinical assessment of neonates still heavily relies on manual procedures and subjective observation (3). Validated tools such as the Neonatal Behavioral Assessment Scale (NBAS) (4), the Test of Infant Motor Performance (TIMP) (5), and the Alberta Infant Motor Scale (AIMS) (6) provide valuable insights into developmental domains and long-term outcomes. However, these tools are time-intensive, require specialized training, and are not typically embedded into routine NICU workflows (7–11). The NAME model differs fundamentally in that it is not intended as a prognostic developmental tool but as a rapid, touch-based assessment to evaluate real-time physiological adaptability in neonates. As such, NAME complements—not replaces—traditional assessments by offering immediate, bedside insights into autonomic and mechanical responsiveness.

In practice, newborns in NICUs are handled between 100 and 200 times per day for various medical and caregiving procedures (8). However, the diagnostic potential of touch remains underutilized. An increasing body of evidence suggests that structured, gentle touch can influence neonatal physiology, particularly through its effects on the autonomic nervous system (ANS), which modulates heart rate variability, oxygen saturation, respiratory patterns, and behavioral states (12–17). Mechanosensory stimulation through low-threshold mechanoreceptors—including Merkel-neurite complexes and C-tactile fibers—has been shown to activate interoceptive brain networks responsible for stress regulation and homeostasis (11, 18–20).

The Neonatal Assessment Manual scorE (NAME) model was developed to operationalize this understanding into a structured bedside tool. NAME evaluates two key parameters: compliance, the infant's global capacity to respond to mechanical stimuli, and homogeneity, the consistency of that response throughout the body (11). The resulting scores are both categorical (“Good,” “Marginal,” “Bad”) and numerical (1–9 Likert scale), allowing for rapid, standardized assessment of an infant's adaptability, which can be completed in under two minutes (11). NAME is designed for interdisciplinary use by trained NICU staff, including nurses, physicians, and therapists. Early research has demonstrated promising validity and reliability metrics (21–23).

However, despite the evidence base, NAME has not yet been integrated into standard clinical routines. As with many novel healthcare innovations, the journey from validation to widespread adoption requires a structured translational framework that bridges the knowledge–practice gap.

To support this process, the present paper adopts the Knowledge-to-Action (KTA) framework, a widely accepted model in implementation science (24). The KTA framework emphasizes a cyclical, pragmatic process through which validated knowledge is synthesized, adapted to local context, and translated into actionable strategies with real-world utility (25). It consists of two interconnected phases: 1) knowledge creation, which involves primary research, synthesis, and development of tools and guidelines; 2) action cycle, which focuses on adapting knowledge to the local context, implementing interventions, monitoring uptake, and sustaining change (24).

In this paper, we apply the KTA framework to translate the NAME model from a validated clinical innovation into a practical, scalable assessment tool for the NICU environment. Specifically, we aim to: a) synthesize and contextualize the current evidence supporting NAME; b) propose a three-tier translational pathway (training, workflow integration, outcome tracking); c) illustrate NAME's bedside utility through a real-world clinical vignette.

We propose that NAME, guided by KTA principles, represents a viable strategy for enhancing neonatal care through physiologically attuned, tactile-based assessment. Its adoption may improve early detection of instability, facilitate interdisciplinary communication, and support precision care planning in the NICU setting.

## 2 Materials and methods

### 2.1 Study design

This manuscript presents a focused narrative synthesis of existing NAME-related studies. Given the limited number of available publications (*n* = 4), all from the same research group, a formal systematic review was not feasible. The authors conducted a targeted literature retrieval using key terms “NAME model”, “neonatal assessment”, and “manual evaluation” across PubMed and Scopus. Due to limited heterogeneity, risk of bias across studies is acknowledged and discussed. This approach prioritizes practical implementation rather than meta-analytic precision. The target audience includes neonatologists, neonatal nurses, manual therapists, and other NICU professionals interested in integrating structured, touch-based assessment tools into daily practice.

The project followed a three-phase structure:

Evidence Synthesis – extraction and appraisal of key findings from foundational NAME research;
Clinical Framework Development – formulation of a stepwise, NICU-compatible implementation model;
Application Modeling – development of a representative clinical vignette to illustrate NAME integration in real care settings.

This methodology aligns with current standards in knowledge translation and clinical implementation science.

### 2.2 Source studies

The translational synthesis was based on four peer-reviewed studies authored by the NAME research team and published between 2020 and 2022 (11, 21–23). These studies were selected because they collectively established the theoretical basis, psychometric validity, reliability, and clinical relevance of the NAME model.

### 2.3 Data extraction and thematic synthesis

Each article was reviewed in full to extract relevant findings on, the physiological rationale behind the NAME model (e.g., ANS activation via structured touch); the structure and function of the NAME assessment procedure; scoring systems and interpretation guidelines; psychometric outcomes, including face/content validity, construct validity, and interrater reliability; clinical correlations with infant gestational age, weight, and complexity indices.

Thematic synthesis was used to categorize and group evidence under three core implementation themes: training, workflow integration, and outcome tracking. This process was informed by implementation science models such as the KTA framework (24, 25) and real-world constraints in neonatal care settings.

### 2.4 Development of the clinical implementation framework

Based on the thematic synthesis, we developed a three-tiered clinical framework for the practical integration of NAME into NICU workflows:

Tier 1: Clinician Training – structured modules on physiological rationale, manual palpation techniques, and score interpretation;

Tier 2: Implementation Flowchart – a step-by-step clinical decision aid for applying NAME assessments during routine NICU rounds and admissions;

Tier 3: Outcome Metrics – recommended process and outcome indicators to evaluate the clinical impact of NAME (e.g., interrater agreement, clinical stratification outcomes, patient course alterations).

This framework was iteratively refined through expert consultation with neonatologists, NICU nurses, and manual therapists familiar with NAME methodology.

### 2.5 Training thresholds

The eligibility thresholds of gestational age ≥29 weeks and birth weight ≥1,000 g were derived from empirical findings in the initial NAME cohort, where intra-rater and inter-rater agreement exceeded 0.85. These cutoffs reflect the feasibility and safety of assessment and optimize reliability by minimizing fragility-related artifacts. While exploratory NAME assessments may be performed in more premature neonates, formal scoring is reserved for those meeting these clinical thresholds.

### 2.6 Clinical use modeling

To further illustrate the translational relevance of NAME, we developed a clinical vignette simulating real-world NICU application. This example was constructed based on anonymized composite data from the original NAME cohort and modeled to demonstrate how NAME scores can inform individualized care strategies, multidisciplinary communication, and longitudinal monitoring.

To ensure the validity of NAME assessments, they are ideally performed when the infant is in a quiet, alert state, with no recent handling, sedation, or acute procedures. These contextual factors are recognized as potential confounders and are considered during training and clinical interpretation.

### 2.7 Ethical considerations

As this work is a secondary synthesis and framework development based on previously published studies, no new data collection involving human subjects was undertaken.

## 3 Results

The results of this translational synthesis are presented in three parts: (1) an in-depth review of the empirical evidence supporting the NAME model (Table 1), (2) the development of a clinically oriented implementation framework, and (3) an illustrative case vignette demonstrating real-world application. Together, these results provide a consolidated foundation for the adoption of NAME into neonatal clinical practice.

**Table 1:** Summary of key evidence from NAME research studies.

Study	Focus area	Sample size	Setting	Assessor background	Operational definitions	Key findings	Confounder control	Implications for practice
Manzotti et al., 2020 (11)	Conceptual development	Not applicable	NICU	Not applicable	Not applicable	Theoretical model based on haptics and interoception; uses compliance and homogeneity	Not applicable	Rationale for structured touch as assessment tool in NICU
Manzotti et al., 2021 (21)	Validity study exploring face, content and construct validity	50 infants: GA: 34.8 ± 3.9wks; BW: 2,181.2 ± 882.5 gr	Single NICU, Italy	2 neonatologists, 1 nurse, 1 physiotherapist, 1 psych- ologist and 5 osteopaths	Complexity index calculated assessing the presence of health complications	High content validity (S-CVI: 0.95); NAME inversely correlated with complexity index (*τ* = −0.31)	No clinical adjustment	NAME discriminates between stable and complicated neonates
Manzotti et al., 2021 (22)	Reliability	144 infants: GA: 35.9 ± 4.1wks; BW: 2,055.3 ± 750.6 gr	Single NICU, Italy	2 physiotherapists/osteopaths	Assessment of both Cohen K and proportion of specific agreements	Moderate-to-good interrater reliability; especially high agreement for ‘Marginal’ scores	Adjusted for sex, GA, BW	Reliable use among trained professionals; adaptable to infant size/age
Manzotti et al., 2022 (23)	Clinical correlation	202 infants: GA: 34.1 ± 4.3 wks; BW: 2,093.4 ± 879.8 gr	Single NICU, Italy	1 neonatologist, 2 physiotherapists/osteopaths	Complexity index calculated assessing the presence and severity of health complications according to the bedside neonatologist	NAME scores correlated with clinical complexity index (OR = 0.84); linked with GA and weight	No clinical adjustment	Supports NAME as early indicator of infant health risk

GA, gestational age; NAME, Neonatal Assessment Manual scorE; NICU, Neonatal Intensive Care Unit; S-CVI, Scale-Content Validity Index; OR, odds ratio; Complexity index definition involved gestational age, birthweight, intrauterine growth restriction, respiratory diseases, cardiovascular pathologies, gastrointestinal pathologies, urogenital diseases, neurological pathologies, metabolic alterations, genetic alterations, surgeries, and other problems (i.e., rare diseases).

### 3.1 Empirical evidence supporting the NAME model

The NAME model is grounded in interoceptive and somatosensory neurophysiology and was designed as a tactile-based evaluation of neonates' physiological adaptability through structured manual palpation (11). A synthesis of four primary studies conducted by Manzotti et al. between 2020 and 2022 provides robust support for NAME's psychometric properties and clinical relevance (11).

#### 3.1.1 Theoretical rationale and model formulation

In the foundational 2020 hypothesis paper, the NAME model was conceptualized as a haptically driven, neurophysiologically informed method of neonatal assessment (11). The authors described the underlying mechanisms by which structured touch may activate low-threshold mechanoreceptors—specifically, C-tactile fibers and Merkel-neurite complexes—resulting in afferent signaling through interoceptive pathways. These inputs, integrated in the anterior insula and limbic structures, modulate the ANS, which governs homeostasis, cardiorespiratory dynamics, and stress responses in neonates. The NAME assessment applies light, static pressure to the cranial and sacral regions, stimulating these afferents and producing a bodily response that is subjectively assessed through two domains: compliance (global responsiveness) and homogeneity (regional consistency of adaptation).

This model introduced a scoring system encompassing both: 1) A categorical score (Good, Marginal, Bad) for rapid clinical interpretation; 2) A numerical score (1–9 scale) to capture more granular physiological changes.

This framework established the foundation for evaluating NAME's clinical utility. Clinical interpretation, indeed, links higher scores with better autonomic regulation and lower complexity indices. NAME therefore provides rapid risk stratification and can inform escalation of care, or the need for targeted monitoring.

#### 3.1.2 Content and construct validity

In 2021, a study involving 18 experienced NICU professionals evaluated the NAME model's validity using expert review and empirical application across 100 neonates (21). The Content Validity Index (CVI) was used to assess the relevance, clarity, and applicability of the NAME procedure and scoring system.

Key findings included: a) High content validity across all items (S-CVI = 0.95), indicating consensus on the clinical appropriateness of NAME; b) Construct validity was demonstrated through a significant inverse correlation between NAME score and a “complexity index” reflecting clinical instability (Kendall's *τ* = –0.31, *p* < 0.01); c) NAME was more likely to classify infants with low clinical complexity as “Good,” and those with higher instability as “Marginal” or “Bad.”

These results support the model's ability to distinguish clinically meaningful differences in neonatal adaptability and condition.

#### 3.1.3 Reliability and inter-rater agreement

The NAME model's reliability was examined in a 2021 interrater agreement study using blinded, paired evaluations from NICU professionals (22). In this study, 120 neonates were assessed independently by two clinicians trained in the NAME procedure. Agreement was measured using Cohen's kappa and weighted statistics.

Key findings that emerged from the study were: a) Moderate-to-good interrater reliability across all score levels (*κ* = 0.56–0.69), with highest agreement for “Marginal” classifications; b) Interrater reliability was positively associated with gestational age and birth weight, suggesting more stable infants yield more interpretable and reproducible tactile responses; c) The model proved usable by multiple operators without a loss of consistency, assuming standard training.

These findings underscore the NAME model's reproducibility and the importance of context-specific calibration.

#### 3.1.4 Clinical correlation in a large neonatal cohort

In the most comprehensive study to date, Manzotti et al. (2022) applied the NAME model to 202 hospitalized neonates of varying gestational ages and clinical profiles (23). This study examined the relationship between NAME scores and several demographic and clinical indicators, including: Gestational age at birth, Birth weight, Neonatal Clinical Complexity Index (NCCI), Postnatal age at assessment. Results demonstrated: a) A statistically significant correlation between higher NAME scores and lower clinical complexity (OR = 0.84, 95% CI: 0.78–0.91; *p* < 0.001); b) Positive associations between NAME scores and gestational age (*p* < 0.01) and birth weight (*p* < 0.01); c) NAME scores were able to reflect subtle gradations in infant condition not captured by routine physiological measurements.

This study provided strong external validation for the NAME model, affirming its sensitivity to clinically relevant variation in neonatal health status.

### 3.2 Translational framework for clinical integration

**Figure 1:**
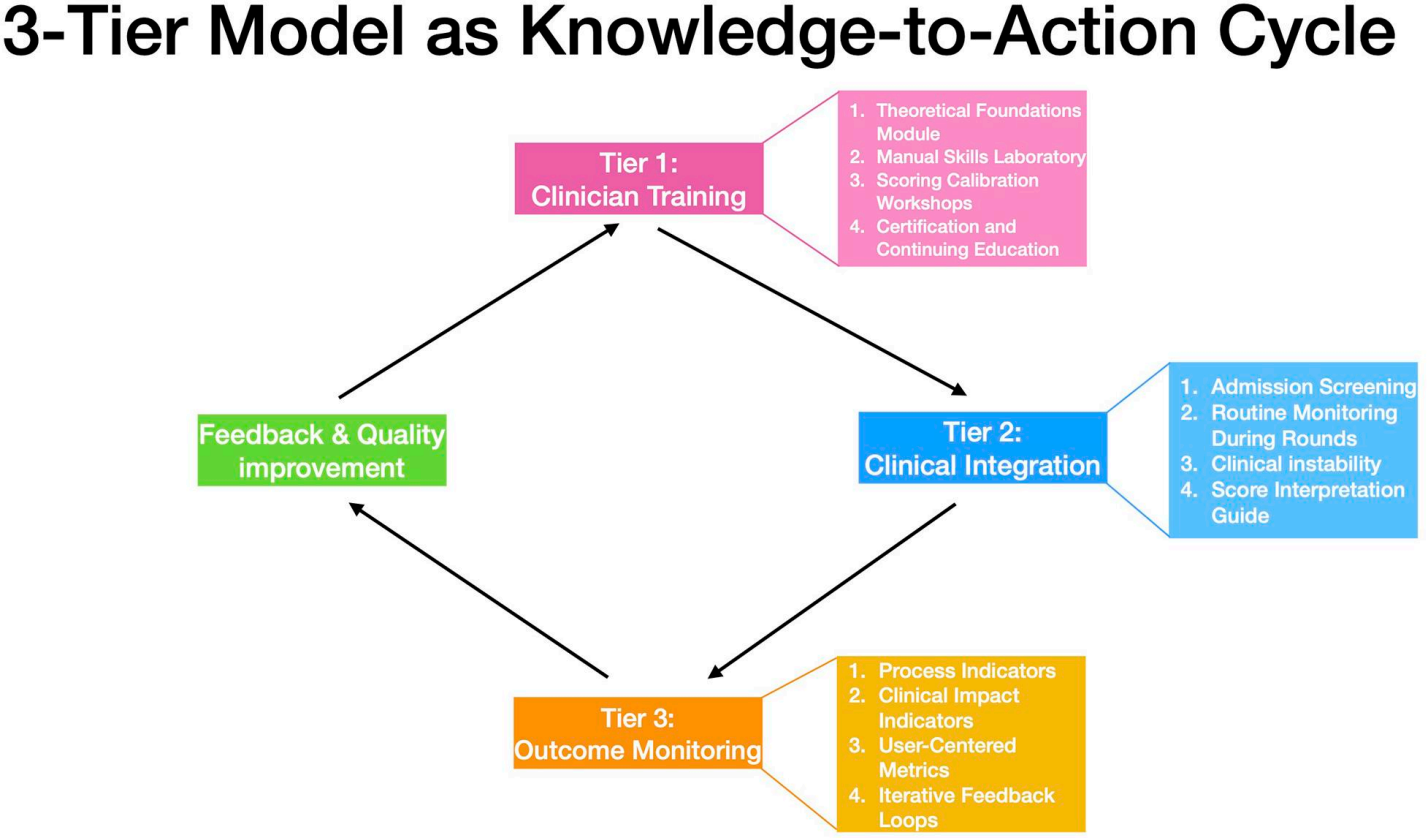
NAME translation via the knowledge-to-action (KTA cycle). A cyclical model aligning the NAME implementation process with the KTA framework. The cycle includes Clinician Training, Clinical Integration, Outcome Monitoring, and iterative Feedback & Quality improvement—facilitating sustainable translation from validated knowledge into practice.

**Figure 2:**
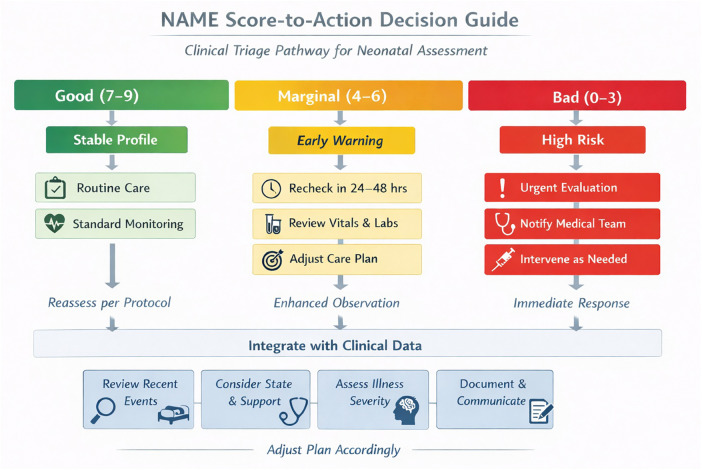
NAME score-to-action decision guide. This flowchart illustrates the clinical integration of the Neonatal Assessment Manual scorE (NAME) model into routine NICU decision-making. Based on the total NAME score (range: 0–9), infants are triaged into three zones: Good (7–9), Marginal (4–6), and Bad (0–3). Each zone corresponds to escalating levels of clinical concern and action planning. Recommended responses range from routine care for stable profiles to urgent evaluation and intervention in high-risk cases. The tool is designed to support, not replace, standard NICU assessments and must be interpreted within the broader clinical context. The lower panel emphasizes integration with clinical data, team communication, and reassessment pathways.

Building on this evidence, a three-tiered translational framework was constructed to support the practical adoption of the NAME model in NICU environments (Figures 1, 2):

Tier 1 – Clinician Training

A structured educational module was proposed, including theoretical background in tactile neurophysiology, hands-on simulation of palpation technique, calibration workshops for scoring reliability, and certification processes.

Tier 2 – Implementation Workflow

A clinical decision pathway was mapped for integrating NAME assessments during: a) NICU admission screening; b) Daily interprofessional rounds; c) High-risk clinical transitions (e.g., escalation, weaning, discharge).

The NAME score informs stratified care responses, from routine monitoring (“Good”) to increased surveillance or multidisciplinary review (“Marginal” or “Bad”).

Tier 3 – Evaluation Metrics

Suggested metrics for evaluating impact include: I) Interrater agreement; II) Changes in care plans triggered by NAME scores; III) Analysis of trajectories, namely of scores varied over time; IV) Associations with clinical outcomes (e.g., length of stay, clinically relevant events); V) Staff satisfaction and usability surveys.

Drawing from the consolidated evidence base of the NAME model and guided by the KTA framework, we propose a three-tier translational model to support the structured implementation of NAME in neonatal intensive care settings. The model addresses key domains of clinician preparation, bedside application, and outcome evaluation. Each tier is designed to ensure that NAME assessments are valid, interpretable, and impactful in daily neonatal care.

#### 3.2.1 Tier 1: clinician training and competency development

The foundation of the NAME implementation framework lies in the structured preparation of clinical staff to ensure fidelity in assessment technique, reliability in scoring, and integration into clinical reasoning (Figure 1, Tier 1 details). Given the model's reliance on haptic sensitivity and nuanced physiological interpretation, a standardized training curriculum is essential to minimize operator variability and ensure intersubjective consistency.

The training program is proposed to be modular and tiered, adapting both in content depth and practical emphasis to match the background and responsibilities of different NICU professionals. It is recommended that the core curriculum include approximately 45–55 h of instruction and practice, divided into didactic and experiential components. Theoretical modules (20–25 h) would provide grounding in neurophysiological principles (including interoception, somatosensory pathways, and autonomic regulation), while practical laboratories (25–30 h) would focus on palpation approaches, clinical simulations, and interrater calibration exercises.

Specific training adaptations by professional profile include:

Neonatologists and Pediatricians: Focus on integrating NAME into diagnostic reasoning, triage decisions, and longitudinal assessment. Emphasis on interpreting trends, understanding physiological implications, and integrating findings into interdisciplinary discussions.

NICU Nurses: Focus on bedside assessment fidelity, routine integration during caregiving tasks, and documenting NAME scores in clinical charts or electronic systems. Particular attention to recognizing changes over time and triggering appropriate team responses.

Manual Practitioners (e.g., osteopaths, physical therapists): Emphasis on tactile proficiency, advanced palpatory skills, and differential interpretation of compliance and homogeneity. Training may include leadership in NAME calibration workshops and support in complex case consultations.

Allied Health Professionals: For roles such as respiratory therapists or psychologists involved in integrated care rounds, optional modules can introduce the conceptual rationale of NAME and its contribution to holistic care planning.

Each participant would undergo competency evaluation through observed structured clinical examinations (OSCEs) or standardized scoring exercises using video scenarios. Certification is granted upon demonstration of consistency (≥80% agreement with reference scores). Refresher sessions and recalibration workshops are advised at 6–12 month intervals to maintain skill fidelity and mitigate scoring drift.

In addition, developing a community of practice within the institution—where NAME-trained staff can engage in joint scoring exercises, reflective discussions, and collaborative case reviews—may enhance both professional engagement and clinical coherence around the tool.

This structured and role-specific approach to training ensures that NAME is not perceived as an isolated tool, but rather as an integrated part of neonatal care culture, adaptable to diverse professional lenses and responsibilities.

#### 3.2.2 Tier 2: clinical workflow integration

The second tier of the implementation framework focuses on embedding the NAME model within the operational and decision-making rhythms of the NICU (Figure 1, Tier 2 details). This tier builds upon the foundational competencies developed during clinician training and translates individual skill into system-wide application. The goal is to ensure that NAME assessments are not isolated events but are consistently and meaningfully integrated into clinical routines, contributing to team-based care and adaptive planning.

The primar*y* axis of this integration involves aligning the NAME assessment with existing care checkpoints. One critical entry point is the neonatal admission phase. During the first 24 to 48 h of hospitalization, every eligible infant—defined as those with a gestational age of at least 29 weeks and a birth weight of 1,000 g or more—is evaluated using the NAME model. This early application provides a physiological baseline that complements traditional vital sign monitoring and supports immediate risk stratification.

Beyond admission, NAME is incorporated into routine monitoring schedules, particularly during interdisciplinary rounds. Conducted at regular intervals (typically every 48 to 72 h), these follow-up assessments allow clinicians to detect subtle changes in autonomic regulation or musculoskeletal tension that may precede overt clinical deterioration. This proactive approach is especially valuable in cases where infants exhibit non-specific signs of stress, which may not be captured through standard metrics alone.

Integration into workflow also includes the use of NAME assessments at critical transition points—such as escalation or de-escalation of respiratory support, initiation of feeding protocols, or prior to discharge planning. These applications help to reinforce the continuity of physiological assessment throughout the infant's care trajectory and promote consistency in interdisciplinary communication.

To ensure interpretability and actionability, the NAME scoring outputs are tied to clinical pathways. A score interpreted as “Good” suggests robust adaptation and stability, warranting continuation of standard care. A “Marginal” score may prompt increased surveillance or the initiation of supportive interventions, such as enhanced tactile interventions, kangaroo care, or physiotherapy consultation. A “Bad” score is treated as a red flag and typically triggers a team-based review, further diagnostic inquiry, and potential escalation of care intensity.

Operational integration also requires digital and administrative alignment. Where possible, NAME scores are recorded within electronic medical records (EMR), enabling trend visualization and integration with broader clinical dashboards. Staff are encouraged to include NAME results in daily handovers and collaborative care discussions, thereby reinforcing a shared physiological language across disciplines.

By positioning NAME within the temporal and procedural architecture of the NICU, Tier 2 transforms the model from a novel assessment tool into a functional and systemic element of care delivery. It ensures that the insights generated through structured touch translate into timely decisions, meaningful interventions, and enhanced continuity of care.

#### 3.2.3 Tier 3: evaluation, feedback, and outcome monitoring

The third tier of the implementation framework focuses on the systematic evaluation of the NAME model's clinical impact and its integration into continuous quality improvement cycles (Figure 1, Tier 3 details). This level is essential for ensuring that NAME not only functions as an individual assessment tool but also contributes meaningfully to the broader clinical, operational, and developmental outcomes of neonatal care.

Outcome evaluation in this tier occurs across three primary dimensions: process fidelity, clinical effectiveness, and user experience. Process fidelity refers to the consistency with which NAME assessments are conducted, recorded, and acted upon. This includes monitoring the frequency of assessments according to protocol, the level of adherence to the scoring algorithm, and the reliability of interpretation across different clinicians. In particular, interrater agreement is continuously measured using standardized cases and blinded parallel scoring sessions, allowing institutions to monitor skill maintenance and scoring drift over time.

Clinical effectiveness encompasses the correlation between NAME scoring and tangible outcomes in neonatal care. Early metrics of interest include changes in care plans prompted by NAME assessments, the alignment of scores with clinical deterioration or recovery events, and the relationship between NAME trends and markers of infant progress—such as stabilization of vital signs, tolerance of feeds, weight gain, and readiness for discharge. These outcome linkages not only validate the physiological sensitivity of the NAME model but also enable the identification of specific thresholds or trajectories that may be predictive of complications or recovery.

Furthermore, NAME outcomes can be correlated with resource utilization indicators, such as duration of respiratory support, frequency of interventions, or length of stay. Through such analyses, the model's value in supporting risk-adjusted decision-making and optimizing care intensity can be quantitatively evaluated. When integrated into institutional dashboards or clinical analytics systems, NAME scores may also serve as early warning indicators or contribute to triage algorithms within EMR platforms.

A third and equally critical aspect of Tier 3 is the evaluation of user experience, both from the perspective of clinicians and families. Structured feedback from staff—captured through surveys, focus groups, or reflective debriefs—can provide insight into the perceived usability, interpretability, and added value of the NAME model in daily practice. This feedback can inform iterative adjustments to training curricula, workflow timing, or documentation (EMR) templates, ensuring that the tool remains practical and relevant.

Notably, this tier also lays the groundwork for incorporating parental feedback and engagement into the evaluation cycle. As proposed in Section 4.4, future development may include parental education on the significance of NAME scores and collaborative care models in which parents are encouraged to participate in regulated touch-based interactions aligned with NAME principles. This co-regulatory model has the potential not only to enhance parental confidence and bonding but to contribute to physiological stabilization and emotional development in the infant.

Critically, Tier 3 adheres to the cyclical logic of the KTA framework by closing the loop between knowledge application and knowledge refinement. The continuous monitoring of performance, outcomes, and contextual feedback allows institutions to adapt implementation strategies, adjust training needs, and revise clinical thresholds based on real-world data. In doing so, the NAME model evolves from a static assessment procedure into a dynamic component of a learning healthcare system—responsive to both individual patient needs and systemic priorities.

### 3.3 Proposed use case in NICU practice

The NAME score can be used:

- Within 24 h of admission to stratify neonatal health status.
- During daily rounds, to track changes and guide intervention decisions.
- As a communication tool, facilitating shared understanding among NICU professionals.

This integrative use promotes early detection of deterioration and individualized care planning—especially for high-risk preterm infants (Figure 2).

### 3.4 Clinical use case vignette: NAME in action

Patient: Baby L., preterm male infant, born at 30 + 2 weeks GA, birth weight 1250g.

Setting: Level III NICU, Day 2 of life.

Clinical Context: Infant stable on non-invasive respiratory support, receiving gavage feeds; no major complications but high baseline risk due to prematurity.

Day 2: Initial NAME Assessment.

**Figure 3:**
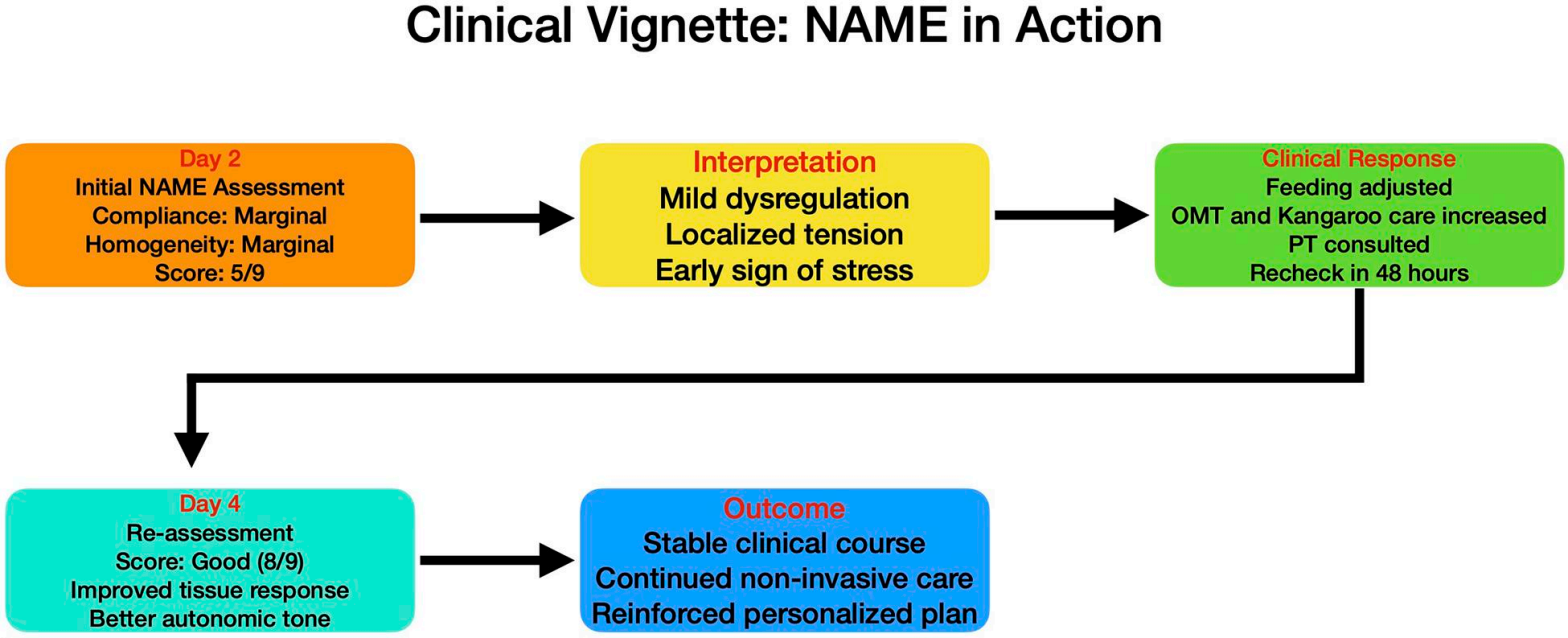
Clinical vignette. This clinical scenario is intended as a hypothetical use case to illustrate the NAME assessment workflow. Interventions such as osteopathic treatment or kangaroo care are context-dependent and should not be interpreted as therapeutic recommendations arising from NAME alone.

Performed during routine morning rounds while infant is in quiet wakefulness (Figure 3).

Compliance: Marginal — body responds to touch but with partial resistance.

Homogeneity: Marginal — increased stiffness noted in lower extremities.

Categorical Score: Marginal.

Numerical Score: 5/9.

Interpretation: Indicates mild dysregulation and localized somatic tension, possibly reflecting early clinical or neurological stress not yet apparent in vitals.

Clinical Response

Neonatologist adjusts feeding position to minimize discomfort. Nurse increases frequency of kangaroo care sessions. Osteopaths consulted for osteopathic assessment and manipulative treatment (OMT). Physical therapist consulted for early neuromotor evaluation. NAME reassessment scheduled in 48 h.

Day 4: Re-assessment.

Categorical Score: Good.

Numerical Score: 8/9.

Outcome: Improved tissue response and homogeneity suggests better ANS regulation. No new complications. Infant continues to thrive.

Impact of NAME Integration.

Enabled early detection of subclinical stress. Promoted interdisciplinary collaboration (nursing, therapy, neonatology). Guided personalized care adjustments based on tactile physiology. Provided objective feedback on infant progress using a non-invasive tool.

This vignette provides an example on how the NAME model can complement traditional metrics (e.g., vitals, labs) by offering physiologically informed, hands-on assessment that enhances both responsiveness and precision in neonatal care planning (Figures 2, 3).

## 4 Discussion

The NAME model represents an innovative and physiologically grounded approach to neonatal assessment that bridges the gap between tactile clinical experience and structured, reproducible evaluation. The model's scientific foundation in interoceptive neurobiology, combined with its demonstrated validity, reliability, and clinical relevance, position it as a promising tool for augmenting neonatal care practices. This discussion synthesizes the evidence for NAME, evaluates its translational potential, compares it with existing neonatal assessment methodologies, and outlines the role of the KTA framework in guiding its implementation.

### 4.1 Evidence-based justification for NAME integration

The findings of this translational synthesis affirm that the NAME model satisfies multiple criteria for clinical utility in the NICU environment. Specifically: i) the model exhibits high content and construct validity, with experts recognizing its clinical relevance (S-CVI = 0.95) and statistical correlations confirming its sensitivity to neonatal complexity and risk status (*τ* = –0.31; OR = 0.84) (21); ii) its reliability profile is suitable for real-world use, with moderate-to-good interrater agreement, especially when performed by trained professionals in stable neonates (22); iii) the model's clinical correlations with gestational age, birth weight, and health trajectories support its use as an early risk stratification tool and longitudinal monitoring aid (23).

These attributes underscore NAME's potential not only as an adjunct to current assessment protocols, but as a unique, rapid-access diagnostic window into autonomic and somatosensory adaptation in the critically ill neonate.

### 4.2 Clinical significance and comparative analysis

Conventional neonatal assessment tools, such as the NBAS, AIMS, and TIMP, are largely developmental in scope and often focus on observable motor and behavioral functions (Table 2). While valuable for long-term follow-up, they are limited in their real-time applicability during acute care phases in the NICU. These tools: a) typically require 10–40 min to administer, b) are highly dependent on infant state and motor readiness, c) require specialized training not universally available in all NICUs (26).

**Table 2:** Comparison of NAME with other neonatal assessment tools.

Tool	Primary modality	Focus	Time to administer	Specialist required	Touch-based?	Clinical usefulness
NAME	Haptic/Tactile	Compliance & homogeneity via ANS response	∼90 s	Manual therapist/NICU-trained staff	Yes	Risk stratification, bedside monitoring
BNBAS (Brazelton)	Visual/Behavioral	Neurobehavioral development	20–30 min	Trained specialist	Limited	Developmental profiling
APIB	Visual/Behavioral	Stress behavior and regulation	25–40 min	Advanced certification	Limited	Research/long-term monitoring
AIMS	Motor observation	Gross motor performance	10–15 min	Physical therapist	No	Motor milestone screening
TIMP	Movement assessment	Functional motor behavior	20–30 min	Therapist/trained clinician	No	Prognostic for motor delay

NAME, Neonatal Assessment Manual scorE; BNBAS, Brazelton Neonatal Behavioral Assessment Scale; APIB, Assessment of Preterm Infant Behavior; AIMS, Alberta Infant Motor Scale; TIMP, Test of Infant Motor Performance. Touch-Based: Defined as requiring structured physical contact as a central part of assessment, beyond handling. Specialist Required: Requires certified personnel with formal training beyond standard NICU roles.

In contrast, the NAME model requires <2 min to perform, offers both categorical and numeric outputs, leverages structured tactile input to evaluate systemic physiological response and aligns closely with the daily manual handling routines already inherent to NICU practice (11). Importantly, the NAME model is not positioned to predict long-term neurodevelopmental outcomes as tools like NBAS or TIMP do. Rather, its strength lies in real-time, physiologically grounded assessment of neonatal adaptability. The tactile and interoceptive focus of NAME captures autonomic tone and mechanical responsiveness, offering clinicians a practical and complementary layer of information during acute care phases.

Additionally, unlike observational-only tools, NAME provides clinicians with an opportunity to interact meaningfully with the infant's regulatory physiology, using gentle touch as both a diagnostic and potentially therapeutic interface. This interoceptive-informed approach aligns with current interest in sensory ecology, trauma-informed neonatal care, and supportive regulation techniques such as kangaroo care and affective touch (27–31).

### 4.3 Clinical integration and impact

Recognizing the implementation gap between validated assessment tools and real-world adoption (32), this paper applied the KTA framework to guide the translation of NAME into clinical practice. The KTA model emphasizes a dynamic and cyclical process of knowledge generation, adaptation, implementation, and evaluation, ensuring innovations are not only evidence-based but context-responsive and sustainably integrated (24, 25).

In alignment with KTA's two-phase structure: i) the Knowledge Creation phase was addressed through the foundational NAME studies, which collectively established theoretical justification, clinical validity, and psychometric rigor; ii) the Action Cycle was addressed through the development of a three-tier implementation framework:

- Clinician Training: Emphasizing tactile proficiency, interrater reliability, and certification;
- Clinical Integration: Mapping NAME use into NICU workflows (e.g., admission assessments, rounds, deterioration points);
- Evaluation Metrics: Tracking process fidelity, clinical impact, and user experience.

The cyclical nature of KTA ensures that NAME's integration is iterative, adaptable to institutional feedback, and capable of evolving in response to clinical realities. Importantly, the model supports scalable adoption across settings, from high-resource tertiary NICUs to lower-resource neonatal units seeking low-cost, high-value clinical assessment options (1, 33).

### 4.4 Strengths, limitations, and future directions

The NAME model offers several compelling strengths that position it as a distinctive addition to neonatal assessment methodologies. Foremost among these is its strong theoretical foundation, rooted in neurophysiology and interoceptive science. Unlike conventional assessment tools that rely predominantly on visual observation or developmental task performance (34, 35), the NAME model is informed by the activation of low-threshold mechanoreceptors and tactile-afferent pathways (19, 36). This biologically grounded approach allows the evaluator to interface directly with the infant's autonomic nervous system through a structured and non-invasive touch protocol.

Another key strength lies in its operational feasibility within high-demand NICU environments. The NAME assessment is designed for brevity, requiring approximately 90 s to complete, and can be performed during routine clinical interactions without disrupting workflow or requiring additional resources. Its dual scoring system—categorical and numerical—supports both rapid clinical triage and longitudinal monitoring of physiological status. Furthermore, the model's compatibility with the existing NICU ecosystem is enhanced by its alignment with core values of neurodevelopmentally supportive care, such as individualized attention, gentle touch, and behavioral regulation (8, 37, 38). This makes it particularly well-suited for integration into multidisciplinary practices involving neonatologists, nurses, physical therapists, and manual practitioners.

Nonetheless, several limitations must be acknowledged to guide both interpretation of current findings and future research directions. While the foundational studies reviewed here provide robust evidence for NAME's validity, reliability, and clinical correlation, they are limited to single-center evaluations conducted within a specific cultural and clinical context. Broader validation across diverse NICU populations, healthcare systems, and staffing models is essential to ensure generalizability and scalability. Additionally, the current evidence base is limited in terms of evaluating NAME's predictive value for specific neonatal morbidities such as sepsis, necrotizing enterocolitis, or intraventricular hemorrhage. While NAME scores reflect dysregulation that may precede these events, future research should investigate whether NAME trends can serve as early markers for such conditions. Until then, NAME should be considered an adjunctive physiological screening tool rather than a diagnostic or prognostic instrument.

Moreover, the potential impact of NAME on clinical decision-making processes, care efficiency, and resource allocation has not yet been quantified. For instance, future research should investigate whether integration of NAME into routine rounds facilitates earlier recognition of deterioration, prompts more timely interventions, or reduces unnecessary escalations. The extent to which NAME contributes to improved communication among team members or enhances parent-clinician engagement through shared understanding of the infant's condition is also worth exploring. These questions are particularly relevant as neonatal care moves toward models emphasizing family-centered, data-informed, and personalized medicine.

A particularly compelling, yet currently underdeveloped, dimension of the NAME model lies in its potential for enhancing parental engagement. Parents of NICU infants often feel disempowered, alienated from their child's care due to the medicalized environment and technological barriers. The NAME model, by virtue of its tactile, relational, and regulatory focus, holds promise as a tool not only for clinicians, but also for parents. Future directions should explore the feasibility and impact of including parents in NAME-informed care frameworks—both as informed observers and as participants in therapeutic touch practices. Introducing structured psycho-soma-education for parents, grounded in NAME's physiological and regulatory framework, could serve dual aims: increasing parental confidence and involvement, and enhancing the infant's neuroregulatory environment through familiar, affective touch. This could be operationalized through guided education sessions, co-regulation approaches, or therapeutic handling workshops facilitated by NICU staff trained in NAME principles.

From an implementation standpoint, it will also be critical to understand the organizational and behavioral enablers or barriers to NAME adoption. Studies exploring clinician perceptions, training sustainability, and institutional readiness will help refine the three-tier framework proposed here. The integration of NAME into EMR systems for automatic score tracking and visualization could further enhance its utility, enabling trend analysis and digital decision support.

Future studies should prioritize prospective, multicenter trials that combine NAME assessments with biomarker data, developmental screening tools, and outcome tracking over time (namely trajectories). Additionally, randomized implementation trials may be valuable in assessing the cost-effectiveness, scalability, and patient-centered impact of NAME in real-world settings. These endeavors will be essential in elevating NAME from a promising, physiologically grounded tool to a standard of care within neonatal medicine.

In summary, while the NAME model exhibits multiple strengths that align with current clinical priorities and care philosophies in neonatology, its full integration into practice will require ongoing research, thoughtful implementation strategies, and a sustained commitment to evidence-informed translation. The framework outlined in this paper, grounded in the Knowledge-to-Action cycle, offers a pathway to support this evolution.

## 5 Conclusions

The Neonatal Assessment Manual scorE model offers a scientifically grounded, physiologically sensitive, and clinically feasible approach to evaluating newborns' adaptive status in the NICU. Synthesizing the findings of four foundational studies, this paper showed that NAME has robust construct validity, interrater reliability, and strong clinical correlations with neonatal health indicators.

By translating NAME into a three-tier clinical framework—comprising structured clinician training, integrated bedside workflow, and measurable evaluation metrics—we provide a roadmap for its adoption in neonatal practice. Unlike conventional assessment tools, NAME harnesses tactile interaction to capture subtle autonomic and mechanical responses, making it uniquely suited to complement current visual and observational assessments.

Its rapid application, safety, and adaptability make NAME a compelling tool for early identification of physiological dysregulation, risk stratification, and longitudinal monitoring. Implementing NAME could enhance clinical decision-making, personalize care strategies, and ultimately improve neonatal health outcomes.

Further studies are warranted to evaluate NAME's predictive capabilities and long-term impact. However, based on current evidence, the NAME model represents a promising advancement in the evolution of neonatal assessment, bridging the gap between physiological insight and clinical practice.

## Data Availability

The original contributions presented in the study are included in the article/Supplementary Material, further inquiries can be directed to the corresponding author.
